# Maternal Chromium Restriction Leads to Glucose Metabolism Imbalance in Mice Offspring through Insulin Signaling and Wnt Signaling Pathways

**DOI:** 10.3390/ijms17101767

**Published:** 2016-10-22

**Authors:** Qian Zhang, Xiaofang Sun, Xinhua Xiao, Jia Zheng, Ming Li, Miao Yu, Fan Ping, Zhixin Wang, Cuijuan Qi, Tong Wang, Xiaojing Wang

**Affiliations:** 1 Key Laboratory of Endocrinology, Translational Medicine Centre, Ministry of Health, Department of Endocrinology, Peking Union Medical College Hospital, Peking Union Medical College, Chinese Academy of Medical Sciences, Beijing 100730, China; zhangqian6088@pumch.cn (Q.Z.); zhengjia_pumch@sohu.com (J.Z.); liming@pumch.cn (M.L.); yumiao@pumch.cn (M.Y.); pingfan@pumch.cn (F.P.); wangzhixinpumch@sohu.com (Z.W.); qicuijuan_pumch@sohu.com (C.Q.); wangtong_pumch@sohu.com (T.W.); wangxiaojingpum@sohu.com (X.W.); 2 Department of Endocrinology, The Affiliated Hospital of Qingdao University, Qingdao 266003, China; sunxiaofang@medmail.com.cn

**Keywords:** development programming, gene expression, glucose metabolism, insulin signaling pathway, Wnt pathway

## Abstract

An adverse intrauterine environment, induced by a chromium-restricted diet, is a potential cause of metabolic disease in adult life. Up to now, the relative mechanism has not been clear. C57BL female mice were time-mated and fed either a control diet (CD), or a chromium-restricted diet (CR) throughout pregnancy and the lactation period. After weaning, some offspring continued the diet diagram (CD-CD or CR-CR), while other offspring were transferred to another diet diagram (CD-CR or CR-CD). At 32 weeks of age, glucose metabolism parameters were measured, and the liver from CR-CD group and CD-CD group was analyzed using a gene array. Quantitative real-time polymerase chain reaction (qPCR) and Western blot were used to verify the result of the gene array. A maternal chromium-restricted diet resulted in obesity, hyperglycemia, hyperinsulinemia, increased area under the curve (AUC) of glucose in oral glucose tolerance testing and homeostasis model assessment of insulin resistance (HOMA-IR). There were 463 genes that differed significantly (>1.5-fold change, *p* < 0.05) between CR-CD offspring (264 up-regulated genes, 199 down-regulated genes) and control offspring. The Kyoto Encyclopedia of Genes and Genomes (KEGG) pathway and STRING (Search Tool for the Retrieval of Interacting Genes/Proteins) analysis revealed that the insulin signaling pathway and Wnt signaling pathway were in the center of the gene network. Our study provides the first evidence that maternal chromium deficiency influences glucose metabolism in pups through the regulation of insulin signaling and Wnt signaling pathways.

## 1. Introduction

Diet has important health effects at any stage of life, but nutrition during fetal and early postnatal life is particularly important for later life, even throughout the whole life. Increasing evidence suggests that the occurrence of diabetes is related to nutrition status in fetus period [1,2,3,4]. Growing evidence proves that under-nutrition in early life is an important risk of metabolic disease in later life [5,6]. The nutrition status of mothers will affect children’s health for a long period. Malnutrition in this sensitive window may lead the occurrence of metabolic disorder in later life [7,8]. Offspring gene changes in this sensitive window are a long-term effect, as they remain in adult life even when dietetic recovery occurs [9]. Damage during this sensitive time will disturb gene expression for a long time.

Besides proteins, fats, and carbohydrates, micronutrients also are necessary for the human body. Although we just need them in trace or tiny amounts daily, some micronutrients (zinc, chromium, magnesium, copper, iron, and potassium) are proven to have an important role in mediation of glucose metabolism. [10]. Chromium can reduce fasting blood glucose levels in diabetic patients and diabetic rodent models [11], and enhance insulin action, including activation of insulin receptor sites [12]. Diabetic animals [13] and patients [14,15] have lower chromium levels than that of controls. Chromium deficiency is common among our population. Chromium levels decrease with advancing age, both in type 2 diabetic patients and normal subjects [16]. A recent study reported that Wistar of National Institute of Nutrition (WNIN) rats born from chromium restriction dams showed insulin resistance and glucose intolerance. Increased oxidative stress was one underlying mechanism [16].

However, no genome-wide study has been conducted to look for key genes and pathways which are affected by maternal chromium restriction in the whole genome. Therefore, we employed the whole genomic microarray to quantify all gene expression in adult mice livers that had maternal exposure to chromium restriction. We hypothesized that there might be some key genes/pathways in the liver which are affected by maternal chromium restriction. The aim of this research is to identify the key genes/pathways in the liver, which are responsible for generating programmed glucose metabolism disorder.

## 2. Results

### 2.1. Dams

At weaning, body weight (22.1 ± 2.5 g vs. 22.8 ± 3.1 g, *n* = 8 per group) and fasting blood glucose (5.9 ± 0.8 mmol/L vs. 6.1 ± 1.2 mmol/L, *n* = 8 per group) in mother mice were not affected by chromium restriction. However, serum chromium concentrations were lower (*p* < 0.01) in the CR (chromium-restricted diet) group (0.33 ± 0.04 ng/mL, *n* = 8 per group) than that in CD (control diet) group (0.75 ± 0.12 ng/mL, *n* = 8 per group).

### 2.2. Pups

#### 2.2.1. Serum Chromium Concentration

**Figure 1:**
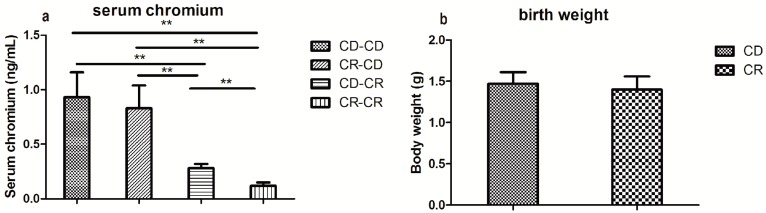

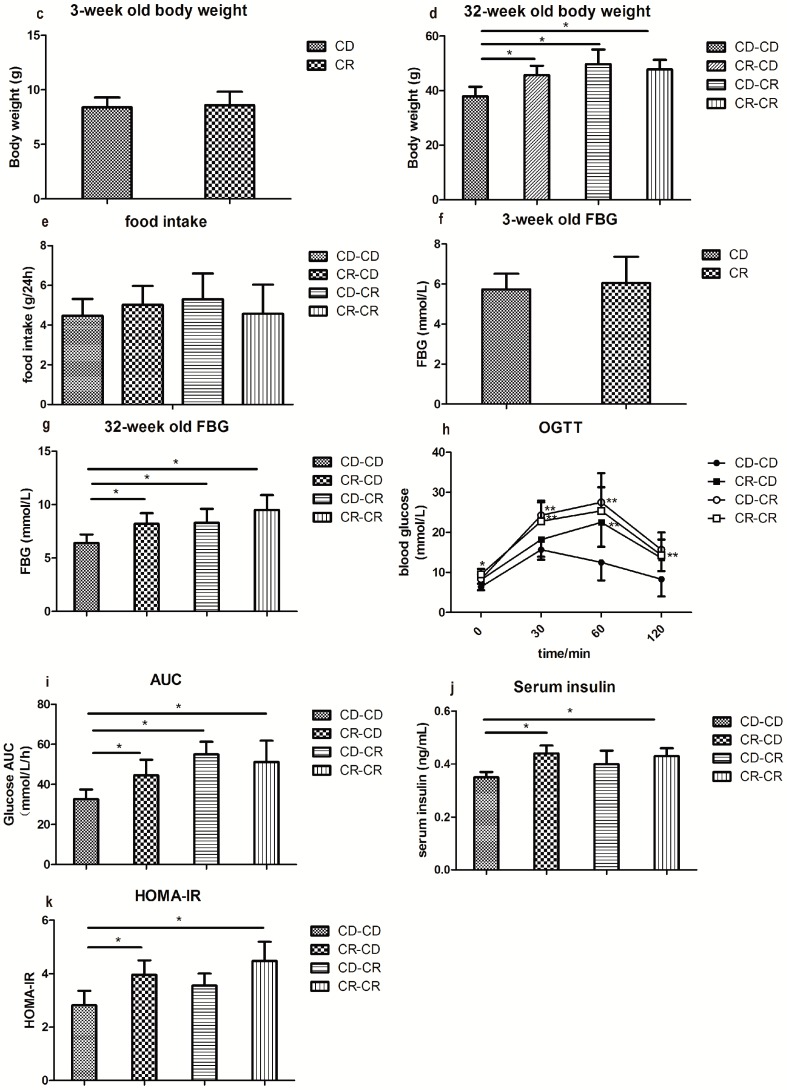
Serum chromium level (**a**) at week 32; body weight on birth day (**b**); week 3 (**c**); and week 32 (**d**); food intake (**e**); fasting blood glucose (FBG) at week 3 (**f**) and week 32 (**g**); and blood glucose in oral glucose tolerance test (OGTT) (**h**) and blood glucose area under the curve (AUC) (**i**) in OGTT, fasting insulin (**j**) and homeostasis model assessment of insulin resistance (HOMA-IR) (**k**) at week 32 in male offspring. CD, control diet; CR: chromium restriction. Each bar represents the mean ± SD. For (**a**,**d**,**e**,**g**–**j**), *n* = 8 per group; for (**b**,**c**,**f**), *n* = 16 per group. * *p* < 0.05, ** *p* < 0.01.

As expected, CD-CR (pups born from control diet dams were fed with chromium restriction diet from weaning) and CR-CR (pups born from chromium restriction dams were fed with chromium restriction diet from weaning) pups had lower serum chromium concentrations than controls (*p* < 0.01, Figure 1a), whereas CR-CD (pups born from chromium restriction dams were fed with control diet from weaning) pups caught up with controls at 32 weeks of age (Figure 1a).

#### 2.2.2. Body Weight and Food Intake

Despite the birth weight and weaning weight being comparable in different groups, at 32 weeks of age, male offspring body weight in CD-CR, CR-CD, and CR-CR groups was higher than the CD-CD (pups born from control diet dams were fed with control diet from weaning) group (*p* < 0.05, Figure 1b–d). However, food intake was comparable among the four groups at 32 weeks of age (Figure 1e).

#### 2.2.3. Fasting Blood Glucose and Glucose Tolerance

In postnatal week 3, fasting blood glucose level was comparable between CR and CD groups (Figure 1f). In postnatal week 32, male CR-CR offspring had significantly higher fasting blood glucose (*p* < 0.05, Figure 1g). The CR-CD regimen could not correct fasting blood glucose to normal levels (*p* < 0.05, Figure 1g). Glucose tolerance was assessed by an oral glucose tolerance test in the offspring at 32 weeks of age. Blood glucose was higher in the CR-CR group and in the CD-CR group before and 30, 60, and 120 min after oral glucose gavage than that in the CD-CD group (*p* < 0.05 or *p* < 0.01, Figure 1h). In the CR-CD group, blood glucose was higher than the CD-CD group before and 60 and 120 min after oral glucose gavage (*p* < 0.05 or *p* < 0.01, Figure 1h). Blood glucose area under the curve (AUC) was higher in CR-CR, CR-CD, and CD-CR groups than the CD-CD group (*p* < 0.05, Figure 1i).

#### 2.2.4. Fasting Insulin and Homeostasis Model Assessment of Insulin Resistance (HOMA-IR)

Fasting insulin and HOMA-IR were higher in the CR-CD group and the CR-CR group than that in the CD-CD group (*p* < 0.05, Figure 1j,k).

#### 2.2.5. Screening of Differentially-Expressed Genes

**Figure 2:**
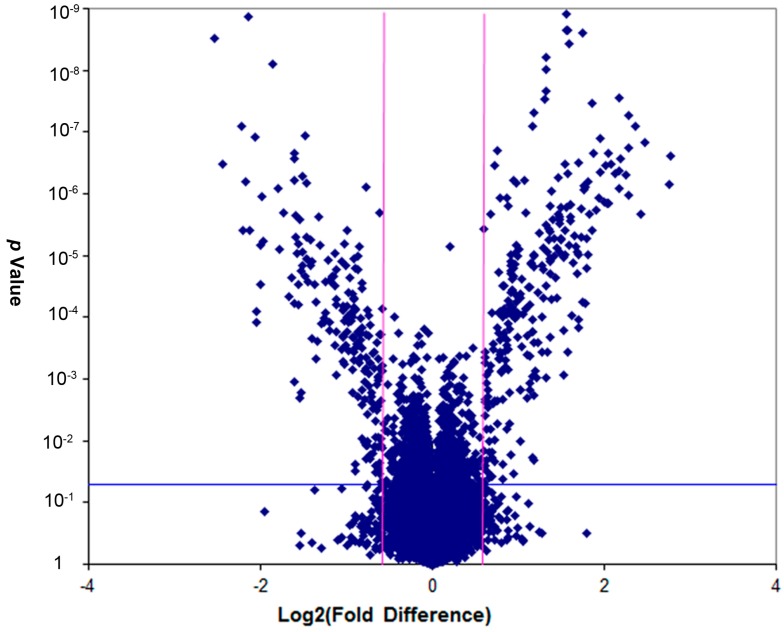
Volcano plot representing the Log2 fold change and the *p*-value of the genes in the array. Volcano plots are a useful tool for visualizing differential expression patterns between two groups. The vertical lines correspond to 1.5-fold up- and down-regulation, and the horizontal line represents a *p*-value of 0.05.

The CR-CD group was fed with a chromium-restricted diet only before weaning. To show the effect of maternal chromium restriction on the gene expression in pup livers, we performed a gene array in the CR-CD and CD-CD groups. Figure 2 shows the gene expression results in the CR-CD group vs. the CD-CD group from the gene array (GEO database: GSE82028, http://www.ncbi.nlm.nih.gov/geo/query/acc.cgi?token=qrqlkmmqztejvqb&acc=GSE82028). We identified 463 significantly differentially-expressed genes, 264 up-regulated genes, and 199 down-regulated genes in the CR-CD group, compared with the CD-CD group (fold change ≥ 1.5, *p* < 0.05). By using hierarchical cluster analysis, differentially expressed genes were clustered into two groups (CR-CD group and CD-CD group). This result suggested that gene expression showed a significant difference between different groups.

#### 2.2.6. Pathway and Gene Ontologies (GO) Analysis

To systematically identify biological connections of differentially-expressed genes and identify new pathways associated with maternal chromium restriction effects on offspring liver, we performed GO and Kyoto Encyclopedia of Genes and Genomes (KEGG) pathway analysis. Data were categorized into three independent gene ontology terms: Biological Process, Cellular Components, and Molecular Function. In the Biological Process ontology, the major GO terms affected in the CR-CD offspring compared with control offspring (*p* < 0.001) were transcription, regulation of transcription, and RNA metabolic process (Table S1). KEGG pathway analysis comparing the CR-CD group against the CD-CD group revealed eight pathways (*p* < 0.001). They are the Wnt signaling pathway, prostate cancer, adherent’s junction, insulin signaling pathway, type 2 diabetes mellitus, endometrial cancer, pathways in cancer, and thyroid cancer (Table S2). Differential expression genes in Wnt signaling and insulin signaling pathway are shown in Figures S1 and S2.

#### 2.2.7. Networks Analysis

By using String online software, 463 differentially expressed genes were mapped in one network (Confidence Score = 400). In this network figure, 269 nodes with 737 joint-edges were included. Forty-six nodes with more than ten joint-edges were considered as important functional molecules in offspring liver affected by maternal chromium restriction (Figures S3 and S4), because they accounted for 87.5% of function in all genes. Of these 46 genes, thymoma viral proto-oncogene 1 (*Akt1*), CREB binding protein (*Crebbp*), catenin beta 1 (*Ctnnb1*), insulin 1 (*Ins1*), cadherin 1 (*Cdh1*), forkhead box O1 (*FoxO1*), androgen receptor (*Ar*), phosphatidylinositol 3-kinase, catalytic, alpha polypeptide (*Pik3ca*), and jun D proto-oncogene (*Jund*) were ranked in the top 10.

#### 2.2.8. Results of Real-Time PCR Verification

**Figure 3:**
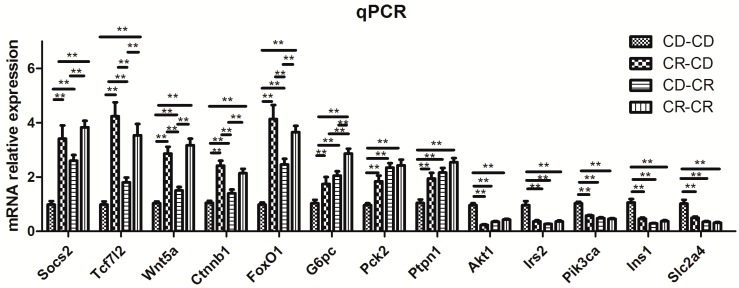
Validation of differentially-expressed genes by real-time PCR. *Socs2*: suppressor of cytokine signaling 2; *Akt1*: thymoma viral proto-oncogene 1; *Irs2*: insulin receptor substrate 2; *Pik3ca*: phosphatidylinositol 3-kinase, catalytic, alpha polypeptide; *Tcf7l2*: transcription factor 7 like 2; *Wnt5a*: wingless-type MMTV integration site family, member 5A; *Ctnnb1* (catenin, beta 1): catenin (cadherin associated protein), beta 1; *FoxO1*: forkhead box O1; *G6pc*: glucose-6-phosphatase, catalytic, *Ins1*: insulin 1; *Pck2* (*Pepck*): phosphoenolpyruvate carboxykinase 2; *Slc2a4* (*Glut4*): solute carrier family 2 member 4; *Ptpn1* *(Ptp1b*): protein tyrosine phosphatase, non-receptor type 1. CD, control diet; CR: chromium restriction. *n* = 8 per group, ** *p* < 0.01.

Since network analysis showed the insulin signaling pathway and Wnt signaling pathway were in the center of all 463 differentially-expressed genes in the CR-CD group compared with the CD-CD group, thirteen differentially-expressed genes in the insulin signaling pathway and Wnt pathway were selected to perform real-time PCR. Real-time PCR results showed that trends in change of expression for the selected 13 genes were consistent with the microarray results. *Socs2*, *Tcf7l2*, *Wnt5a*, *Ctnnb1* (catenin, beta1), *G6pc*, *Pck2* (*Pepck*), *FoxO1*, and *Ptpn1* (*Ptp1b*) showed higher expression levels, and lower expression in *Akt1*, *Irs2*, *Pik3ca*, *Slc2a4* (*Glut4*), and *Ins1* in the CR-CD, CD-CR, and CR-CR groups than that in the CD-CD group (Figure 3, *p* < 0.01).

#### 2.2.9. Western Blot

**Figure 4:**
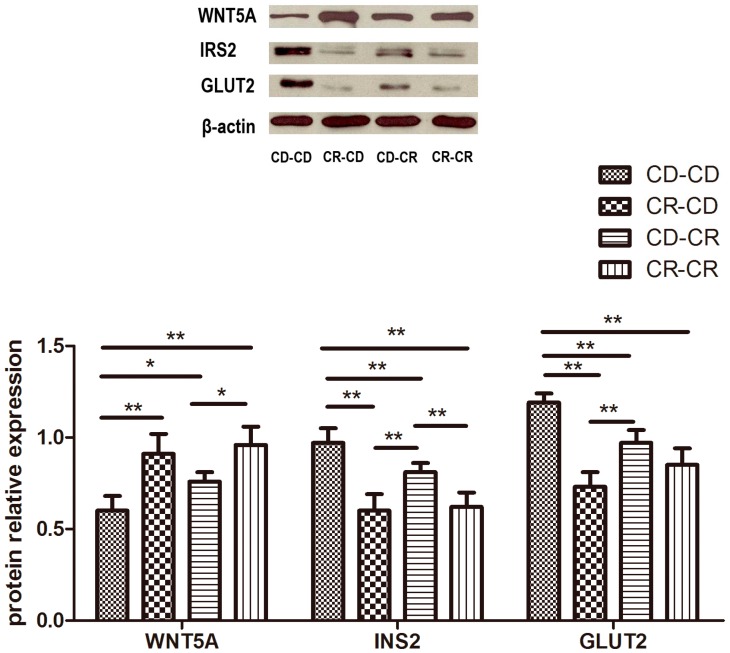
The protein levels were detected by Western blot. IRS2: insulin receptor substrate 2; SLC2A2 (GLUT2): solute carrier family 2 member 2; WNT5A: wingless-type MMTV integration site family, member 5A. CD, control diet; CR: chromium restriction. *n* = 8 per group, * *p* < 0.05, ** *p* < 0.01.

Total protein expression of IRS2 and SLC2A2 (GLUT2) was down-regulated, while WNT5A was up-regulated more significantly in the CR-CD, CD-CR, and CR-CR groups than in the CD-CD group (*p* < 0.01, Figure 4).

## 3. Discussion

In this study, a chromium-restricted diet excluded 90% of chromium potassium sulfate from the control diet (0.14 mg chromium/kg diet). The adequate intake for a human as recommended by the National Research Council is 30 μg chromium/day. Although the dose we used is higher than the human recommended minimum dose, this dose is the lowest chromium content in rodent diets. Our results showed that a 29-week chromium-restricted diet (CD-CR group) led to obesity and hyperglycemia in a mouse model. Previous studies revealed the key role of chromium as a blood glucose and lipid regulator [17,18]. Both in a diabetic rodent model and patients, chromium supplementation could improve glucose and lipid metabolism [11,19,20,21].

More importantly, we found that maternal chromium restriction (CR-CR group and CR-CD group) did not change the birth weight of offspring. However, maternal chromium restriction increased offspring body weight at 32 weeks of age. Even the body weight of CR-CD group, which was converted to normal diet after weaning and had normal serum chromium levels, was higher than the CD-CD group. These results demonstrated that a maternal chromium-restricted diet irreversibly increased body weight. In other maternal undernutrition models, other scientists found similar results. Kumar et al. found that offspring born to vitamin-B12 deficiency dams had a lower birth weight and higher body weight from three months [22], and we found that the food intake among four groups were comparable. This finding suggests that hyperphagia may not be the cause of obesity, but differences in basal energy expenditure may contribute.

In addition, we found that maternal chromium restriction irreversibly led to glucose intolerance in 32-week-old offspring. Inagadapa et al. also found that maternal chromium restriction increased fasting plasma glucose (from nine months), and the area under the curve of glucose during oral glucose tolerance testing (from 15 months) in the offspring [16]. In our study, the mice from chromium-restricted diet dams showed glucose metabolism disorder earlier than in Inagadapa’s results, which is partly due to the lower chromium content that we used. Other maternal undernutrition diets, such as maternal protein restriction, also leads offspring to impaired glucose tolerance [23].

Moreover, maternal chromium restriction irreversibly increased fasting insulin levels and HOMA-IR. Previous study also found that maternal chromium restriction increased fasting insulin (from 15 months) and HOMA-IR (from nine months). Rehabilitation could not correct this effect [16]. Other maternal mineral-restricted models also showed hyperinsulinemia and insulin resistance, such as magnesium restriction. Moreover, pups which switched to the control diet from the magnesium restriction diet after lactation or weaning had even higher serum insulin and higher HOMA-IR [24]. From our results, mice from maternal chromium restriction diets showed higher body weight and insulin resistance. We cannot dismiss the contribution of obesity to insulin resistance. However, from Inagadapa’s study, the early development of insulin resistance (from nine months) in male rats from maternal chromium restriction could promote metabolic disorders that lead to increased adipose tissue (from 18 months).

By using the STRING gene network and KEGG pathway analysis, we found that some genes in the insulin signaling pathway were in the center of the gene function network. Real-time PCR results showed that a maternal chromium restriction diet can inhibit the insulin signaling pathway, such as insulin receptor substrate 2 (*Irs2*), phosphatidylinositol 3-kinase catalytic alpha polypeptide (*Pi3kca*), and thymoma viral ptoto-oncogene (*Akt1*). Moreover, the total protein expression of IRS2 and GLUT2 was also down-regulated by maternal chromium restriction. In the liver, insulin promotes glucose uptake and suppresses hepatic glucose production by stimulating a cascade of signaling processes initiated by the binding of insulin to extracellular α-subunit of the insulin receptor (IR) on the cellular membrane. In the presence of insulin, IR phosphorylates insulin receptor substrate (IRS), which are linked to the activation of the PI3K-Akt pathway, which is responsible for most of the metabolic actions of insulin [25]. Ye et al. found hepatocytes in intrauterine growth restriction (IUGR) rats with catch-up growth showing decreased *Irs1* and *Pi3k* expression [26]. Chromium plays a vital role in glucose regulation in vivo. Chromium picolinate can stimulate phosphorylation of IRS-1 and PI3K activity in the skeletal muscle of an insulin-resistant rat model, JCR: LA-corpulent rat [27]. Chromium (d-Pheylalanine)_3_ enhances insulin-stimulated phosphorylation of Akt in a time- and concentration-dependent manner in adipocytes of ob/ob mice [28]. Our previous study also shows chromium-containing drugs can improve blood glucose through activating insulin signaling [29].

Otherwise, we found maternal chromium restriction could activate the expression of *G6pc*, *Pck2* (*Pepck*), and *FoxO1* in offspring liver. FoxO1 can be phosphorylated through the activation of Akt. Phosphorylated FoxO1 became inactivated. Thus, the phosphorylation of FoxO1 by Akt reduces its entry into the nucleus. In the nucleus, FoxO1 could regulate the expression of *G6pc* and *Pck2,* which are two key enzymes in hepatic gluconeogenesis [30]. In our study, maternal chromium restriction impaired PI3K-Akt signaling, thus activating *FoxO1*, resulting in excess hepatic glucose production. A maternal high-fat diet increased FOXO1 in islets of adult mice offspring [31]. A maternal vitamin D-restricted diet activated *FoxO1* in offspring islets [32]. Thus, our study showed that maternal chromium restriction could stimulate hepatic glucose production in the livers of pups, contributing to the fasting hyperglycemia.

Another interesting finding is that a maternal chromium restriction diet could increase the suppressor of cytokine signaling 2 (*Socs2*) expression in the liver. The SOCS family can inhibit cytokine-induced signaling pathway. They can also be a part of classical negative feedback loop [33]. The expression of SOCSs are upregulated in liver, skeletal muscle, and adipose tissue in insulin resistance animal model [34,35]. More importantly, SOCSs can regulate insulin signaling through mediating several molecules, such as IR and IRS [36]. Transferring *Socs3* siRNA in hepatocytes from IUGR rats with catch-up growth might mediate insulin signaling disorder [26].

Moreover, in KEGG pathway analysis, Wnt signaling is at the topmost rank. We found that mRNA expression of *Ctnnb1* (β-catenin), *Tcf7l2*, and *Wnt5a*, and the total protein expression of WNT5A, is up-regulated by maternal chromium restriction. The Wnt/β-catenin pathway has a key role in embryonic development, cellular fate and migration, tumorigenesis, and organogenesis [37]. Wnt ligands can activate β-catenin through binding to membrane receptors. Activated β-catenin transfers into nucleus. In the nucleus, β-catenin binds to T cell factor (TCF) to regulate the gene expression. One cohort study in Japanese population found a single nucleotide polymorphism (SNP) site in *Wnt5b* gene is correlative with risk of diabetes [38]. Transcription factor 7-like 2 (*Tcf7l2*) is a unique gene which is a type 2 diabetes candidate gene in the TCF family [39]. More and more studies highlight the role of the Wnt pathway in regulating glucose metabolism in several different models. Wnt signaling may alter the activity of the kinase of Akt, thereby modulating insulin signal transduction [40]. Liu et al. found that acute liver-specific deletion of β-catenin resulted in lower fasting plasma glucose concentrations and improved glucose tolerance in high-fat diet mice [41]. Thus, maternal chromium restriction could activate Wnt signaling in the livers of offspring to disturb the glucose metabolism.

## 4. Materials and Methods

### 4.1. Study Design and Animals

All procedures were performed with the approval of the Animal Care Committee of the Peking Union Medical Hospital Animal Ethics Committee (Project identification code: MC-07-6004, 15 March 2013), and all efforts were made to minimize suffering. Seven-week-old female and male C57BL mice (18.4 ± 1.3 g) were purchased from the Institute of Laboratory Animal Science, Chinese Academy of Medical Sciences and Peking Union Medical College (Beijing, China, SCXK-2013-0107). All mice were maintained in individual cages in a room at 24 ± 1 °C with lights on from 6:00 a.m. to 6:00 p.m. Mating was performed by housing females with adult males overnight, and pregnancy was confirmed by examining vaginal smears for the presence of sperm. After mating, the females were randomly divided into two groups and fed either the control diet (CD, casein-based diet based on the American Institute of Nutrition AIN-93G diet, containing 1.19 mg chromium/kg diet) or a chromium-restricted diet (CR, only reduced by 90 percent of chromium potassium sulfate from the control diet, containing 0.14 mg chromium/kg diet). The concentration of chromium in the diet was analyzed by an atomic absorption spectrometer (TAS986, Beijing Persee General Corperation, Beijing, China). All diets were produced by Research Diets (New Brunswick, NJ, USA). Mice were fed with this diet during the pregnancy and lactation periods. After the birth of the offspring, the litter size in each cage was randomly adjusted to six pups (three males, three females, if possible) to ensure equal nutrition until the pups were weaned.

**Figure 5:**
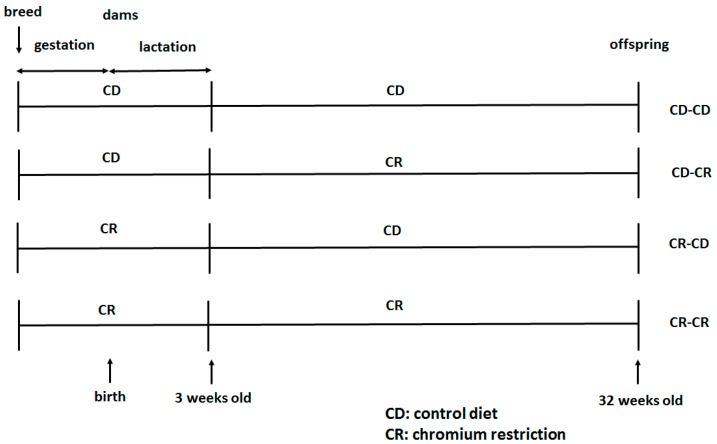
Schematic representation of the feeding protocol. CD, control diet; CR: chromium restriction.

After weaning, offspring were separated into four groups: CD-CD group, CD-CR group, CR-CD group, and CR-CR group. Only male offspring were used for the present study to avoid sex differences on the effect of maternal nutrition on glucose metabolism [42]. At the end of the experimental period (32 weeks of age), male mice (*n* = 8 per group) were sacrificed. A blood sample was collected from the intraorbital retrobulbar plexus from the anesthetized mice (10 h fasted, ketamine 100 mg/kg i.p., Pharmacia and Upjohn Ltd., Crawley, UK). Livers of the offspring were quickly removed and stored at −80 °C for further analysis. Figure 5 shows the feeding protocol.

### 4.2. Measurement of the Serum Chromium Level

Serum was obtained from the dams (*n* = 8 per group) at weaning and offspring (*n* = 8 per group) at eight months of age for chromium analysis. The blood samples were collected in serum-separated tubes to obtain serum. The coagulated blood was left to clot at room temperature for 30 min and then centrifuged for 15 min at 2000 rpm at 4 °C. The supernatant fluid was then separated and stored frozen (−80 °C) for analysis. For chromium determination, serum was diluted at a ratio of 1:4 in 0.1% Triton-X 100 and 0.01 mol/L nitric acid. All laboratory glassware was soaked by nitric acid and rinsed with deionized water to avoid metal contamination. The serum chromium concentration was measured by an atomic absorption spectrometer (Atomic Absorption Spectrophotometer, Hitachi, Japan). Chromium standard solution for the calibration curve (0, 0.1, 0.2, 0.5, 1, 2 ng/mL Cr) was freshly prepared by serial dilution of the stock solution (100 ng/mL Cr) with 0.01 mol/L nitric acid.

### 4.3. Measurements of Body Weight and Food Intake

Body weight of dams were measured at weaning. At birth, weaning and age of 32 weeks, all of the pups were weighed. At 32 weeks of age, food intake was determined for each group by weighing the total amount of food given at the start of the week and then subtracting the amount of food remaining at the end of the week. The average food consumed per mouse was then obtained by dividing by the number of mice.

### 4.4. Fasting Blood Glucose, Oral Glucose Tolerance Test (OGTT)

Blood glucose measurements from tail blood were recorded using a blood glucose analyzer (Bayer Contour TS glucometer, Bayer, Hamburg, Germany). Oral glucose tolerance test was performed after fasting of the mice overnight (32 weeks old). After collection of fasting sample (time 0), 2.0 g of glucose/body weight was administered, and tail blood samples were collected at 30, 60, and 120 min after glucose administration. The area under the curve (AUC) was calculated for blood glucose (BG) during the OGTT using the following equation:

$$\mathrm{AUC}=0.5\times \frac{BG0+BG30}{2}+0.5\times \frac{BG30+BG60}{2}+1\times \frac{BG60+BG120}{2}$$

### 4.5. Measurement of Serum Insulin and HOMA-IR

Mice were food-deprived overnight and serum was obtained to analyze insulin (ELISA, Mouse insulin kit, Millipore, Bellerica, MA, USA) concentration according to the manufacturer’s protocols. Insulin resistance was assessed from fasting plasma glucose and insulin concentrations by computing the homeostasis model assessment of insulin resistance (HOMA-IR) values, according to the following formula:

$$\text{HOMA}-\text{IR}=\frac{Fasting\;insulin\;\left(\mu IU/mL\right)\;\times \;Fasting\;glucose\;\left(mmol/L\right)}{22.5}$$

### 4.6. RNA Extraction from Liver and Whole Liver Genome Expression Profiling

RNA was extracted from 250 mg of mice livers using an RNA Isolation Kit (mirVanaTM, Ambion, San Paulo, Brazil). RNA quantity and quality were assessed by Nanodrop ND-1000 (Nanodrop, Wilmington, DE, USA). The 260/280 ratios of all samples were between 1.95 and 2.10. The integrity and quality of the RNA was assessed using the Bioanalyzer (Agilent Technologies, Santa Clara, CA, USA). All RNA integrity number (RIN) values were ≥7.3. To synthesize cDNA, 10 μL total RNA was mixed with 4 μL Master Mix, 2 μL specific primers, and completed up to 20 μL with H_2_O. The cycling conditions were: 25 °C for 5 min, 42 °C for 30 min, and 85 °C for 5 min. Then, the Affymetrix GeneChip Mouse Gene 2.0 ST whole transcript-based array (Affymetrix, Santa Clara, CA, USA) covering 26,515 genes, was used to analyze mRNA expression. Three microarrays each were performed from the CD-CD group and the CR-CD group. All arrays were uploaded into Genespring v 12.5 (Agilent Tchnologies, Palo Alto, CA, USA) for data normalization, quality control, and first-pass analysis. Differentially-expressed genes were then identified through fold change, as well as *p*-value, calculated with a *t*-test. The threshold set for up- and down-regulated genes was a fold change ≥1.5 and a *p*-value ≤0.05.

### 4.7. Pathway and Network Analysis

To clarify the function of differentially expressed genes and involved pathway, bioinformatics analysis was performed. By using DAVID functional analysis tool (Database for Annotation, Visualization, and Integrated Discovery [43], Leidos Biomedical Research Inc., Frederick, MD, USA), differentially expressed genes were enriched into different gene ontologies (GO) [44] and Kyoto Encyclopedia of Genes and Genomes (KEGG) pathways [45]. Moreover, STRING Software (Biobyte Solution, Heidelberg, Germany) [46] analyzed the connections among differentially expressed genes in involved pathway.

### 4.8. Real-Time PCR Validation

Real-time PCR analysis of gene expression was performed in an ABI Prism 7500 system (Applied Biosystems, Foster City, CA, USA). The primers used were obtained from Applied Biosystems (Table S3). Target mRNA expression was normalized to CypA expression and expressed as a relative value using the comparative threshold cycle (*C*_t_) method (2^−ΔΔ*C*t^) according to the manufacturer’s instructions.

### 4.9. Western Blot

Protein samples were prepared with lysis buffer (1% Triton X-100, 150 mmol.L NaCl, 10 mmol/L Tris-HCL, pH 7.4, 1 mmol/L EDTA, 1 mmol/L EGTA, pH 8.0, 0.2 mmol/L Na_3_VO_4_, 0.2 mmol/L phenylmethysulfonyl fluoride, and 0.5% NP-40). Lysates were centrifuged (13,000 rpm, 4 °C for 10 min), and protein was quantified using Bradford staining (Bio-Rad, Hercules, CA, USA). Equal amounts (50 μg per sample) of protein from each sample was applied to 10% sodium dodecyl sulfate polyacrylamide gel electrophoresis (SDS-PAGE) and electrotransferred to polyvinylidene difluoride (PVDF) membranes (Bio-Rad, Hercules, CA, USA), and then blocked in 5% skimmed milk dissolved in Tris-buffered saline buffer (TBS; 10 mmol/L Tris, pH 7.5, 100 mmol/L NaCl) overnight at 4 °C with the primary antibodies. The expression levels of IRS2 (IRS2 rabbit antibody, 1:1000, Cell Signaling Technology, Danvers, MA, USA), GLUT2 (GLUT2 rabbit antibody, 1:1000, Cell Signaling Technology, Danvers, MA, USA), WNT5A (WNT5A rabbit antibody, 1:1000, Cell Signaling Technology, Danvers, MA, USA), and β-actin (as internal control, β-actin rabbit antibody, 1:1000, Abcam, Cambidge, MA, USA) were determined using specific antibodies. Appropriated secondary antibodies conjugated to horseradish peroxidase (1:3000, Cell Signaling Technology, Danvers, MA, USA) were incubated with respective membranes for 2 h at room temperature. This was followed by five intermittent washes with 1× TBS buffer. Visualization was performed with the use of enhanced chemiluminescence (ECL; Cell Signaling Technology, Danvers, MA, USA). The results were quantified by densitometric analysis using Image-J software (NIH, National Institute of Health, Bethesda, MD, USA). The experiments were replicated three times.

### 4.10. Data Analysis

The data are expressed as the mean and the associated standard deviation of the mean. Differences between the groups were analyzed using one of the following tests: unpaired Student’s *t*-test, or one-way ANOVA followed by Tukey’s post hoc test. A *p*-value ≤0.05 was considered statistically significant (Prism version 5.0 for Windows, GraphPad Software, San Diego, CA, USA).

## 5. Conclusions

**Figure 6:**
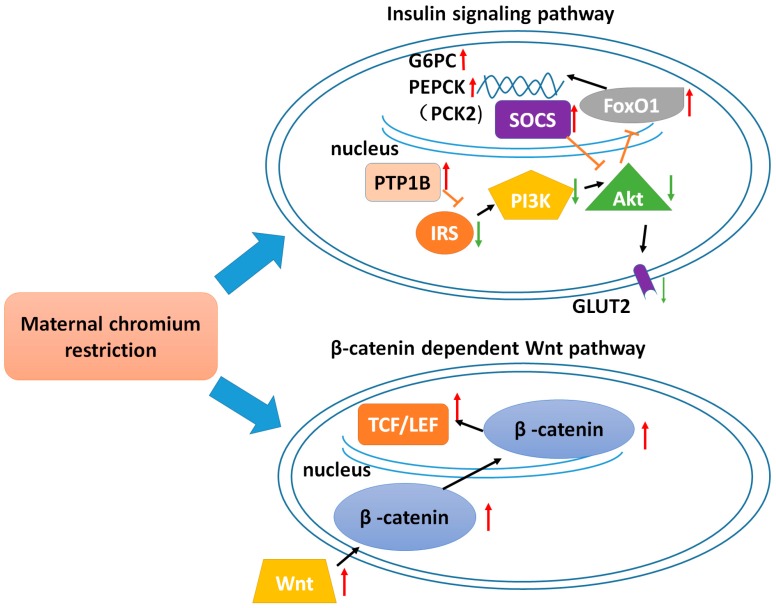
Schematic representation of maternal chromium restriction on the insulin signaling pathway and β-catenin-dependent Wnt pathway. In the Wnt pathway, maternal chromium restriction activates offspring liver β-catenin through Wnt, resulting β-catenin enters the nucleus. In nucleus, β-catenin binds to T cell factor/lymphoid enhancer factor (TCF/LEF) to activate target genes. In the insulin signaling pathway, maternal chromium restriction inhibits insulin receptor substrate (IRS)/phosphatidylinositol 3-kinase (PI3K)/Akt/protein kinase B (PKB) signaling pathway, enhancing the expression of G6PC and PEPCK to increase hepatic glucose production. Black arrows show the interaction of genes. T–bars show the inhibition of genes. Red arrows show upregulation of genes. Green arrows show downregulation of genes. SOCS: suppressor of cytokine signaling; Akt: thymoma viral proto-oncogene; IRS: insulin receptor substrate; PI3K: phosphatidylinositol 3-kinase; TCF/LEF: transcription factor/lymphoid enhancer factor; FOXO1: forkhead box O1; G6PC: glucose-6-phosphatase, catalytic; GLUT2: glucose transporter 2; PTP1B: protein tyrosine phosphatase, non-receptor type 1.

In summary, our data shows that adult mice from chromium-restricted dams display obesity, hyperglycemia, and insulin resistance. More importantly, the insulin signaling pathway and Wnt signaling pathway may be the underlying mechanisms that lead to glucose metabolism disturbance (Figure 6). These results are important for the understanding of the glucose metabolism disorder caused by chromium restriction during the embryonic and suckling period. Our study points to the need for prevention of diabetes and the consumption of chromium-restricted diets during this important period for the development of the glucose metabolic balance in later life. Novel drugs targeting insulin signaling and Wnt signaling pathways are expected to prevent and intervene in the glucose metabolism disease programming to reduce the global burden of diabetes.

## Supplementary Materials

Supplementary materials can be found at www.mdpi.com/1422-0067/17/10/1767/s1.

## Abbreviations

AUC	the area under the curve
GO	gene ontologies
HOMA-IR	homeostasis model assessment of insulin resistance
KEGG	Kyoto Encyclopedia of Genes and Genomes
OGTT	oral glucose tolerance test
RIN	RNA integrity number

## References

[1] Whincup P.H., Kaye S.J., Owen C.G., Huxley R., Cook D.G., Anazawa S., Barrett-Connor E., Bhargava S.K., Birgisdottir B.E., Carlsson S. (2008). Birth weight and risk of type 2 diabetes: A systematic review. JAMA.

[2] Vaag A.A., Grunnet L.G., Arora G.P., Brons C. (2012). The thrifty phenotype hypothesis revisited. Diabetologia.

[3] Hales C.N., Barker D.J., Clark P.M., Cox L.J., Fall C., Osmond C., Winter P.D. (1991). Fetal and infant growth and impaired glucose tolerance at age 64. BMJ.

[4] Molendi-Coste O., Laborie C., Scarpa M.C., Montel V., Vieau D., Breton C. (2009). Maternal perinatal undernutrition alters postnatal development of chromaffin cells in the male rat adrenal medulla. Neuroendocrinology.

[5] Kimani-Murage E.W., Kahn K., Pettifor J.M., Tollman S.M., Dunger D.B., Gomez-Olive X.F., Norris S.A. (2010). The prevalence of stunting, overweight and obesity, and metabolic disease risk in rural south african children. BMC Public Health.

[6] Sawaya A.L., Roberts S. (2003). Stunting and future risk of obesity: Principal physiological mechanisms. Cadernos de Saúde Pública.

[7] Lesage J., Sebaai N., Leonhardt M., Dutriez-Casteloot I., Breton C., Deloof S., Vieau D. (2006). Perinatal maternal undernutrition programs the offspring hypothalamo-pituitary-adrenal (HPA) axis. Stress.

[8] Moura A.S., Carpinelli A.R., Barbosa F.B., Gravena C., Mathias P.C. (1996). Undernutrition during early lactation as an alternative model to study the onset of diabetes mellitus type II. Res. Commun. Mol. Pathol. Pharmacol..

[9] De Oliveira J.C., Scomparin D.X., Andreazzi A.E., Branco R.C., Martins A.G., Gravena C., Grassiolli S., Rinaldi W., Barbosa F.B., Mathias P.C. (2011). Metabolic imprinting by maternal protein malnourishment impairs vagal activity in adult rats. J. Neuroendocrinol..

[10] Candilish D.J. (2000). Minerals. J. Am. Coll. Nutr..

[11] Feng W., Mao G., Li Q., Wang W., Chen Y., Zhao T., Li F., Zou Y., Wu H., Yang L. (2015). Effects of chromium malate on glycometabolism, glycometabolism-related enzyme levels and lipid metabolism in type 2 diabetic rats: A dose-response and curative effects study. J. Diabetes Investig..

[12] Rhodes N.R., McAdory D., Love S., Di Bona K.R., Chen Y., Ansorge K., Hira J., Kern N., Kent J., Lara P. (2010). Urinary chromium loss associated with diabetes is offset by increases in absorption. J. Inorg. Biochem..

[13] Morris B.W., Kemp G.J., Hardisty C.A. (1985). Plasma chromium and chromium excretion in diabetes. Clin. Chem..

[14] Morris B.W., MacNeil S., Hardisty C.A., Heller S., Burgin C., Gray T.A. (1999). Chromium homeostasis in patients with type II (NIDDM) diabetes. J. Trace Elem. Med. Biol..

[15] Rajendran K., Manikandan S., Nair L.D., Karuthodiyil R., Vijayarajan N., Gnanasekar R., Kapil V.V., Mohamed A.S. (2015). Serum chromium levels in type 2 diabetic patients and its association with glycaemic control. J. Clin. Diagn. Res..

[16] Padmavathi I.J., Rao K.R., Raghunath M. (2011). Impact of maternal chromium restriction on glucose tolerance, plasma insulin and oxidative stress in wnin rat offspring. J. Mol. Endocrinol..

[17] Krol E., Krejpcio Z. (2010). Chromium(III) propionate complex supplementation improves carbohydrate metabolism in insulin-resistance rat model. Food Chem. Toxicol..

[18] Hao C., Hao J., Wang W., Han Z., Li G., Zhang L., Zhao X., Yu G. (2011). Insulin sensitizing effects of oligomannuronate-chromium (III) complexes in C2C12 skeletal muscle cells. PLoS ONE.

[19] Feng W., Zhao T., Mao G., Wang W., Feng Y., Li F., Zheng D., Wu H., Jin D., Yang L. (2015). Type 2 diabetic rats on diet supplemented with chromium malate show improved glycometabolism, glycometabolism-related enzyme levels and lipid metabolism. PLoS ONE.

[20] Racek J., Sindberg C.D., Moesgaard S., Mainz J., Fabry J., Muller L., Racova K. (2013). Effect of chromium-enriched yeast on fasting plasma glucose, glycated haemoglobin and serum lipid levels in patients with type 2 diabetes mellitus treated with insulin. Biol. Trace Elem. Res..

[21] Onakpoya I., Posadzki P., Ernst E. (2013). Chromium supplementation in overweight and obesity: A systematic review and meta-analysis of randomized clinical trials. Obes. Rev..

[22] Kumar K.A., Lalitha A., Pavithra D., Padmavathi I.J., Ganeshan M., Rao K.R., Venu L., Balakrishna N., Shanker N.H., Reddy S.U. (2013). Maternal dietary folate and/or vitamin B_12_ restrictions alter body composition (adiposity) and lipid metabolism in wistar rat offspring. J. Nutr. Biochem..

[23] Vo T.X., Revesz A., Sohi G., Ma N., Hardy D.B. (2013). Maternal protein restriction leads to enhanced hepatic gluconeogenic gene expression in adult male rat offspring due to impaired expression of the liver X receptor. J. Endocrinol..

[24] Venu L., Kishore Y.D., Raghunath M. (2005). Maternal and perinatal magnesium restriction predisposes rat pups to insulin resistance and glucose intolerance. J. Nutr..

[25] Taniguchi C.M., Emanuelli B., Kahn C.R. (2006). Critical nodes in signalling pathways: Insights into insulin action. Nat. Rev. Mol. Cell Biol..

[26] Ye J., Zheng R., Wang Q., Liao L., Ying Y., Lu H., Cianflone K., Ning Q., Luo X. (2012). Downregulating SOCS3 with sirna ameliorates insulin signaling and glucose metabolism in hepatocytes of iugr rats with catch-up growth. Pediatr. Res..

[27] Wang Z.Q., Zhang X.H., Russell J.C., Hulver M., Cefalu W.T. (2006). Chromium picolinate enhances skeletal muscle cellular insulin signaling in vivo in obese, insulin-resistant JCR:LA-cp rats. J. Nutr..

[28] Yang X., Li S.Y., Dong F., Ren J., Sreejayan N. (2006). Insulin-sensitizing and cholesterol-lowering effects of chromium (d-phenylalanine)_3_. J. Inorg. Biochem..

[29] Zhang Q., Xiao X.H., Li M., Li W.H., Yu M., Zhang H.B., Ping F., Wang Z.X., Zheng J. (2014). Chromium-containing traditional Chinese medicine, Tianmai Xiaoke tablet improves blood glucose through activating insulin-signaling pathway and inhibiting PTP1B and PCK2 in diabetic rats. J. Integr. Med..

[30] Krebs M., Roden M. (2005). Molecular mechanisms of lipid-induced insulin resistance in muscle, liver and vasculature. Diabetes Obes. Metab..

[31] Bringhenti I., Ornellas F., Mandarim-de-Lacerda C.A., Aguila M.B. (2016). The insulin-signaling pathway of the pancreatic islet is impaired in adult mice offspring of mothers fed a high-fat diet. Nutrition.

[32] Maia-Ceciliano T.C., Barreto-Vianna A.R., Barbosa-da-Silva S., Aguila M.B., Faria T.S., Mandarim-de-Lacerda C.A. (2016). Maternal vitamin D-restricted diet has consequences in the formation of pancreatic islet/insulin-signaling in the adult offspring of mice. Endocrine.

[33] Krebs D.L., Hilton D.J. (2000). SOCS: Physiological suppressors of cytokine signaling. J. Cell Sci..

[34] Emanuelli B., Peraldi P., Filloux C., Chavey C., Freidinger K., Hilton D.J., Hotamisligil G.S., van Obberghen E. (2001). SOCS-3 inhibits insulin signaling and is up-regulated in response to tumor necrosis factor-alpha in the adipose tissue of obese mice. J. Biol. Chem..

[35] Ueki K., Kondo T., Kahn C.R. (2004). Suppressor of cytokine signaling 1 (SOCS-1) and SOCS-3 cause insulin resistance through inhibition of tyrosine phosphorylation of insulin receptor substrate proteins by discrete mechanisms. Mol. Cell. Biol..

[36] Lebrun P., van Obberghen E. (2008). SOCS proteins causing trouble in insulin action. Acta Physiol..

[37] Clevers H. (2006). Wnt/β-catenin signaling in development and disease. Cell.

[38] Kanazawa A., Tsukada S., Sekine A., Tsunoda T., Takahashi A., Kashiwagi A., Tanaka Y., Babazono T., Matsuda M., Kaku K. (2004). Association of the gene encoding wingless-type mammary tumor virus integration-site family member 5B (*WNT5B*) with type 2 diabetes. Am. J. Hum. Genet..

[39] Neve B., Le Bacquer O., Caron S., Huyvaert M., Leloire A., Poulain-Godefroy O., Lecoeur C., Pattou F., Staels B., Froguel P. (2014). Alternative human liver transcripts of TCF7L2 bind to the gluconeogenesis regulator HNF4α at the protein level. Diabetologia.

[40] Abiola M., Favier M., Christodoulou-Vafeiadou E., Pichard A.L., Martelly I., Guillet-Deniau I. (2009). Activation of wnt/beta-catenin signaling increases insulin sensitivity through a reciprocal regulation of wnt10b and srebp-1c in skeletal muscle cells. PLoS ONE.

[41] Liu H., Fergusson M.M., Wu J.J., Rovira I.I., Liu J., Gavrilova O., Lu T., Bao J., Han D., Sack M.N. (2011). Wnt signaling regulates hepatic metabolism. Sci. Signal..

[42] Yokomizo H., Inoguchi T., Sonoda N., Sakaki Y., Maeda Y., Inoue T., Hirata E., Takei R., Ikeda N., Fujii M. (2014). Maternal high-fat diet induces insulin resistance and deterioration of pancreatic β-cell function in adult offspring with sex differences in mice. Am. J. Physiol. Endocrinol. Metab..

[43] Dennis G., Sherman B.T., Hosack D.A., Yang J., Gao W., Lane H.C., Lempicki R.A. (2003). David: Database for annotation, visualization, and integrated discovery. Genome Biol..

[44] Ashburner M., Ball C.A., Blake J.A., Botstein D., Butler H., Cherry J.M., Davis A.P., Dolinski K., Dwight S.S., Eppig J.T. (2000). Gene ontology: Tool for the unification of biology. The gene ontology consortium. Nat. Genet..

[45] Kanehisa M., Araki M., Goto S., Hattori M., Hirakawa M., Itoh M., Katayama T., Kawashima S., Okuda S., Tokimatsu T. (2008). Kegg for linking genomes to life and the environment. Nucleic Acids Res..

[46] Szklarczyk D., Franceschini A., Wyder S., Forslund K., Heller D., Huerta-Cepas J., Simonovic M., Roth A., Santos A., Tsafou K.P. (2015). STRING v10: Protein-protein interaction networks, integrated over the tree of life. Nucleic Acids Res..

