# Supplementary material for: Maternal Chromium Restriction Leads to Glucose Metabolism Imbalance in Mice Offspring through Insulin Signaling and Wnt Signaling Pathways
Source: Int J Mol Sci. 2016 Oct 22;17(10):1767. doi: 10.3390/ijms17101767 (PMC5085791; doi:10.3390/ijms17101767)
Supplement: Supplementary file 1 [file ijms-17-01767-s001.pdf]

Supplementary Materials: Maternal Chromium Restriction Leads to Glucose Metabolism Imbalance in Mice Offspring through Insulin Signaling and Wnt Signaling Pathways

Qian Zhang, Xiaofang Sun, Xinhua Xiao, Jia Zheng, Ming Li, Miao Yu, Fan Ping, Zhixin Wang, Cuijuan Qi, Tong Wang and Xiaojing Wang

Table S1. The most enrichment GO terms ($p < 0.001$).

Term	Count	Fold Enrichment	p-Value	Catalogue
transcription	60	1.8330	3.07×10^{-6}	Biology Process
regulation of transcription	70	1.7016	4.52×10^{-6}	Biology Process
regulation of RNA metabolic process	51	1.8554	1.66×10^{-5}	Biology Process
regulation of transcription, DNA-dependent	50	1.8476	2.32×10^{-5}	Biology Process
positive regulation of transcription, DNA-dependent	21	2.7328	8.75×10^{-5}	Biology Process
positive regulation of RNA metabolic process	21	2.7132	9.71×10^{-5}	Biology Process
response to insulin stimulus	8	7.3404	9.90×10^{-5}	Biology Process
positive regulation of transcription from RNA polymerase II promoter	19	2.8731	1.14×10^{-4}	Biology Process
insulin receptor signaling pathway	6	11.6004	1.41×10^{-4}	Biology Process
regulation of transcription from RNA polymerase II promoter	26	2.2849	1.76×10^{-4}	Biology Process
positive regulation of nitrogen compound metabolic process	23	2.3671	2.91×10^{-4}	Biology Process
tube development	15	3.0758	3.88×10^{-4}	Biology Process
positive regulation of nucleobase, nucleoside, nucleotide and nucleic acid metabolic process	22	2.3352	4.87×10^{-4}	Biology Process
positive regulation of transcription	21	2.3933	5.05×10^{-4}	Biology Process
positive regulation of cellular biosynthetic process	23	2.2556	5.60×10^{-4}	Biology Process
positive regulation of biosynthetic process	23	2.2353	6.34×10^{-4}	Biology Process
positive regulation of gene expression	21	2.3296	7.06×10^{-4}	Biology Process
ventricular cardiac muscle cell differentiation	4	19.6852	9.10×10^{-4}	Biology Process
nucleoplasm part	21	2.4608	3.34×10^{-4}	Cellular Components
nucleoplasm	23	2.3082	3.84×10^{-4}	Cellular Components
transcription regulator activity	46	2.2728	2.00×10^{-7}	Molecular Function
DNA binding	55	1.8401	6.91×10^{-6}	Molecular Function
transcription factor activity	29	2.2268	9.76×10^{-5}	Molecular Function
zinc ion binding	58	1.6418	9.96×10^{-5}	Molecular Function
transition metal ion binding	68	1.5536	1.01×10^{-4}	Molecular Function
transcription factor binding	15	3.1035	3.51×10^{-4}	Molecular Function

Table S2. Most enrichment pathways by KEGG ($p < 0.001$).

Pathway ID	Pathway	Count	Genes	Fold Enrichment	*p*-Value
mmu04310	Wnt signaling pathway	15	*Wnt5a*, *Ppp2r1a*, *Tcf7*, *Ppard*, *Ppp2r5b*, *Apc2*, *Crebbp*, *Skp1a*, *Tcf7l2*, *Porcn*, *Tcf7l1*, *Ctnnb1*, *Rac3*, *Ppp3cb*, *Frat1*	4.5484	1.81×10^{-5}
mmu05215	Prostate cancer	11	*Akt1*, *Tcf7*, *Ar*, *Creb3*, *Crebbp*, *Foxo1*, *Pik3ca*, *Ins1*, *Pik3r3*, *Tcf7l2*, *Tcf7l1*	5.5659	2.20×10^{-5}
mmu04520	Adherens junction	10	*Tcf7*, *Rac3*, *Crebbp*, *Lmo7*, *Cdh1*, *Ptpn1*, *Insr*, *Tcf7l2*, *Iqgap1*, *Tcf7l1*	5.9920	3.45×10^{-5}
mmu04910	Insulin signaling pathway	13	*Akt1*, *Irs2*, *Socs2*, *Slc2a4*, *Inpp5k*, *Tsc2*, *Foxo1*, *Pik3ca*, *Ptpn1*, *Ins1*, *Pik3r3*, *Pck2*, *Insr*	4.2899	4.00×10^{-5}
mmu04930	Type II diabetes mellitus	8	*Irs2*, *Socs2*, *Slc2a4*, *Cacna1g*, *Pik3ca*, *Ins1*, *Pik3r3*, *Insr*	7.4350	7.90×10^{-5}
mmu05213	Endometrial cancer	8	*Akt1*, *Tcf7*, *Apc2*, *Pik3ca*, *Cdh1*, *Pik3r3*, *Tcf7l2*, *Tcf7l1*	7.0061	1.17×10^{-4}
mmu05200	Pathways in cancer	19	*Wnt5a*, *Ar*, *Ppard*, *Tcf7*, *Apc2*, *Rxra*, *Crebbp*, *Itga2*, *Foxo1*, *Cdh1*, *Tcf7l2*, *Tcf7l1*, *Arnt*, *Akt1*, *Ccdc6*, *Rac3*, *Pik3ca*, *Rarb*, *Pik3r3*	2.6788	1.92×10^{-4}
mmu05216	Thyroid cancer	6	*Ccdc6*, *Tcf7*, *Rxra*, *Cdh1*, *Tcf7l2*, *Tcf7l1*	9.4220	3.53×10^{-4}

Table S3. Real-time PCR primers.

Transcripts	ID	Primer Direction	Primer Sequence	Product Length
Socs2	NM_007706	F	5′-TTAGAGATAGTTCGCATTCAGA-3′	79
		R	5′-TCAATCCGCAGGTTAGTC-3′	
Akt1	NM_009652	F	5′-CAGTTTGAGACCACACAT-3′	75
		R	5′-GCGTCAGTCCTTAATAGTT-3′	
Irs2	NM_001081212	F	5′-AGGACTATGGAGACATTGAG-3′	75
		R	5′-GCATGTAGCCATCATCTG-3′	
Pik3ca	NM_008839	F	5′-TGTGGCATCTGAGTATCT-3′	132
		R	5′-TGTGGCATCTGAGTATCT-3′	
Tcf7l2	NM_001142918	F	5′-GGACACCACTCAACAATG-3′	76
		R	5′-TACAGCAAGCAACCTCTAA-3′	
Wnt5a	NM_009524	F	5′-GAGTATCGTCCTTCATTGC-3′	101
		R	5′-TCCTGGTATTCGTGGTAG-3′	
Ctnnb1 (catenin, beta 1)	NM_007614	F	5′-ATGGAGGAGATAGTAGAA-3′	98
		R	5′-AATGGAATGGTATTGAGT-3′	
FoxO1	NM_019739	F	5′-CCTTGAGCAGCCTAATGT-3′	108
		R	5′-CGATGGACGGAATGAGAG-3′	
G6pc	NM_008061	F	5′-GAAGGATGGAGGAAGGAA-3′	77
		R	5′-TTGGTAATTCACTTGGAGATAG-3′	
Ins1	NM_008386	F	5′-GGAGGTACTTTGGACTAT-3′	79
		R	5′-GATGGTCTCTGATTATAGC-3′	
Pck2 (Pepck)	NM_028994	F	5′-CCTCTGACAATCGTGCTA-3′	128
		R	5′-GTTATTATGTTCAATCCAACTCTG-3′	
Slc2a4 (Glut4)	NM_009204	F	5′-TATGTTGCGGATGCTATG-3′	82
		R	5′-TTAGGAAGGTGAAGATGAAG-3′	
Ptpn1 (Ptp1b)	NM_011201	F	5′-CCACGAACAGAGTCTAAT-3′	79
		R	5′-TGAGAGCAAGAAGAGTATT-3′	

Socs2: suppressor of cytokine signaling 2; *Akt1*: Thymoma viral proto-oncogene 1; *Irs2*: insulin receptor substrate 2; *Pik3ca*: phosphatidylinositol 3-kinase, catalytic, alpha polypeptide; *Tcf7l2*: transcription factor 7 like 2; *Wnt5a*: wingless-type MMTV integration site family, member 5A; *Ctnnb1* (catenin, beta 1): catenin (cadherin associated protein), beta 1; *FoxO1*: forkhead box O1; *G6pc*: glucose-6-phosphatase, catalytic, *Ins1*: insulin 1; *Pck2* (*Pepck*): phosphoenolpyruvate carboxykinase 2; *Slc2a4* (*Glut4*): solute carrier family 2 member 4; *Ptpn1* (*Ptp1b*): protein tyrosine phosphatase, non-receptor type 1.

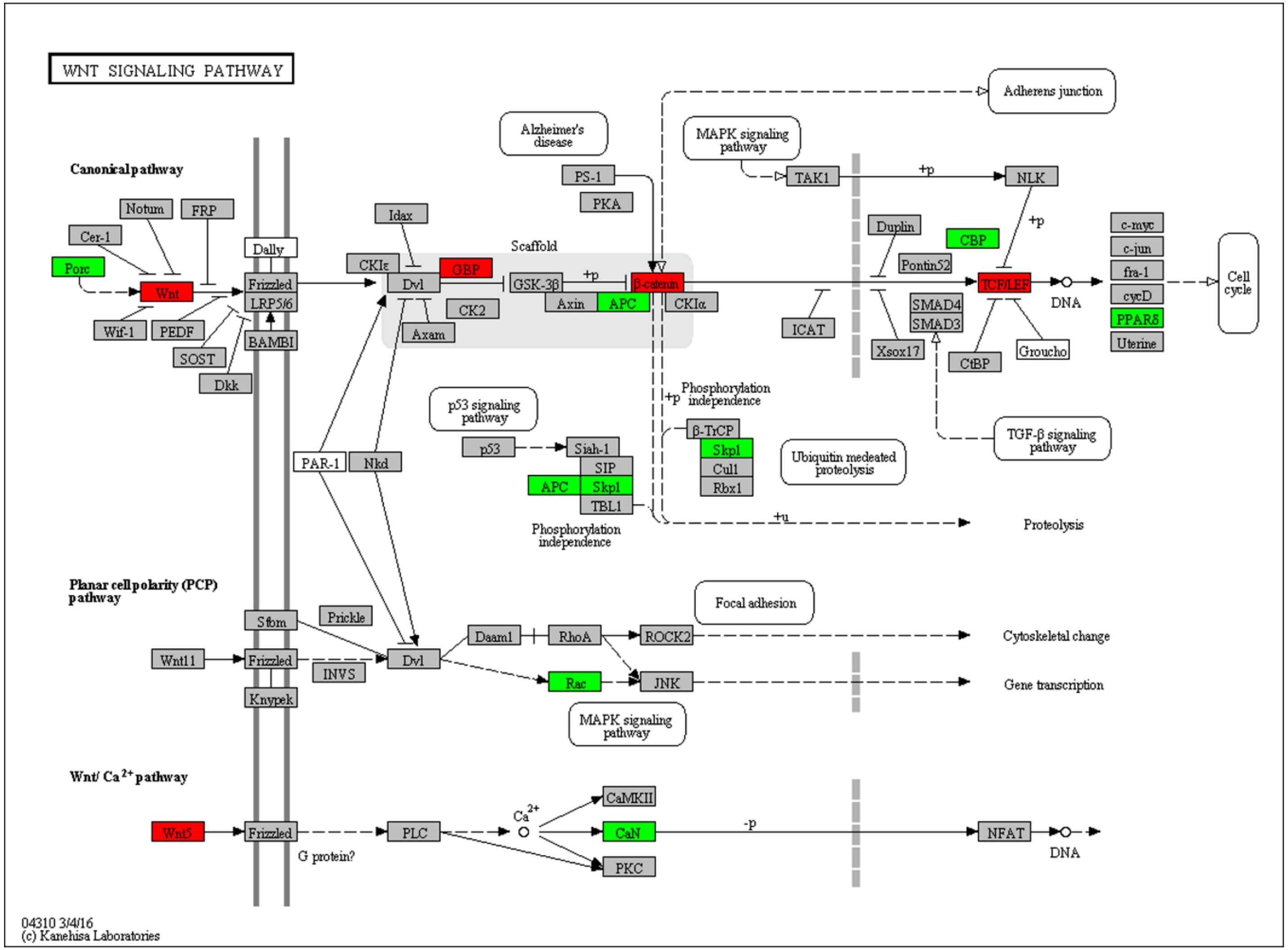

Figure S1. Wnt pathway. Red represents up-regulated; green represents down-regulated; grey represents no significant change. Arrows with dashed line show possible interaction of genes. Arrows with solid line show interaction of genes. T bars show inhibition of genes.

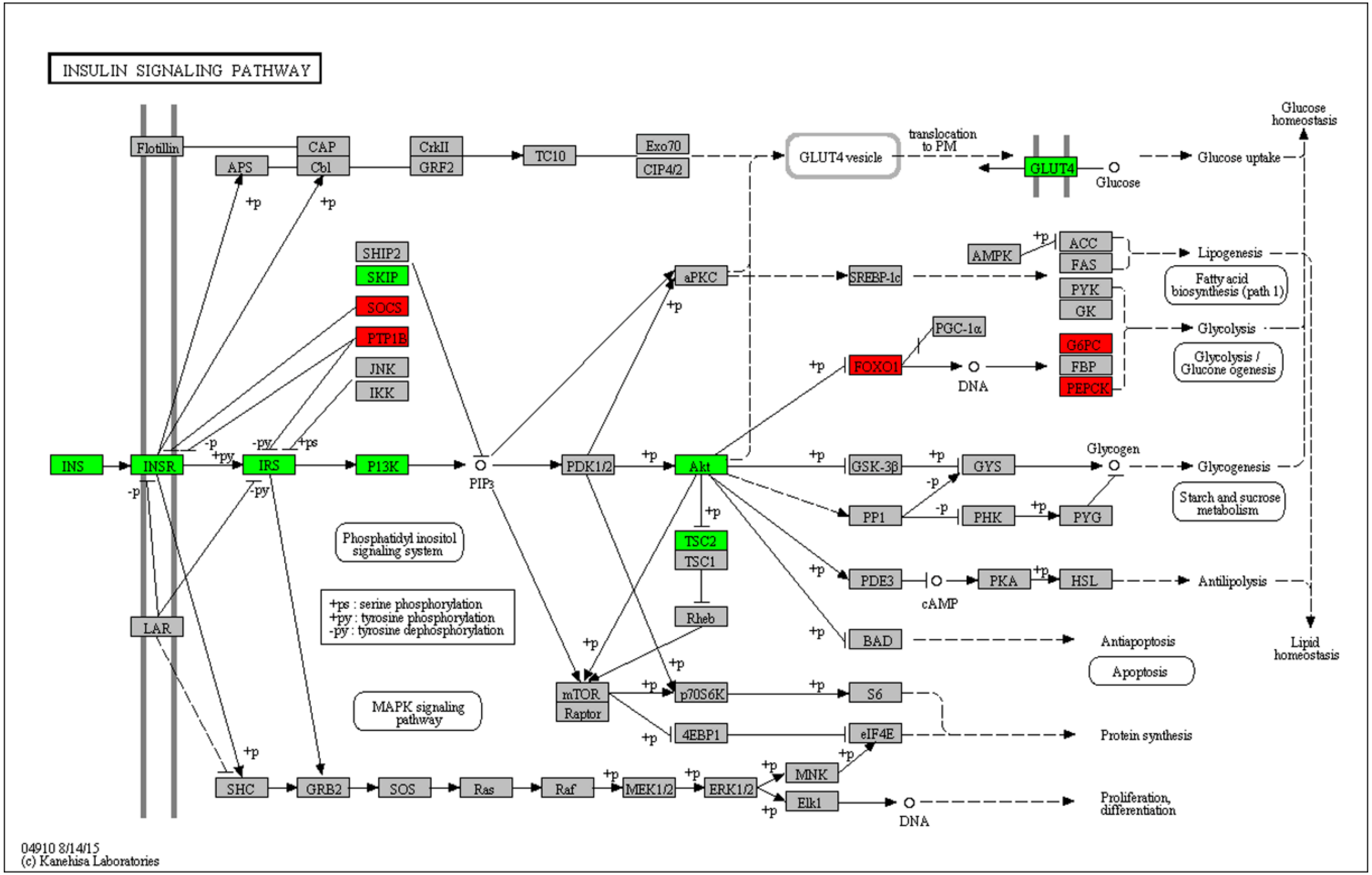

Figure S2. Insulin signaling pathway. Red represents up-regulated; green represents down-regulated; grey represents no significant change. Arrows with dashed line show possible interaction of genes. Arrows with solid line show interaction of genes. T bars show inhibition of genes.

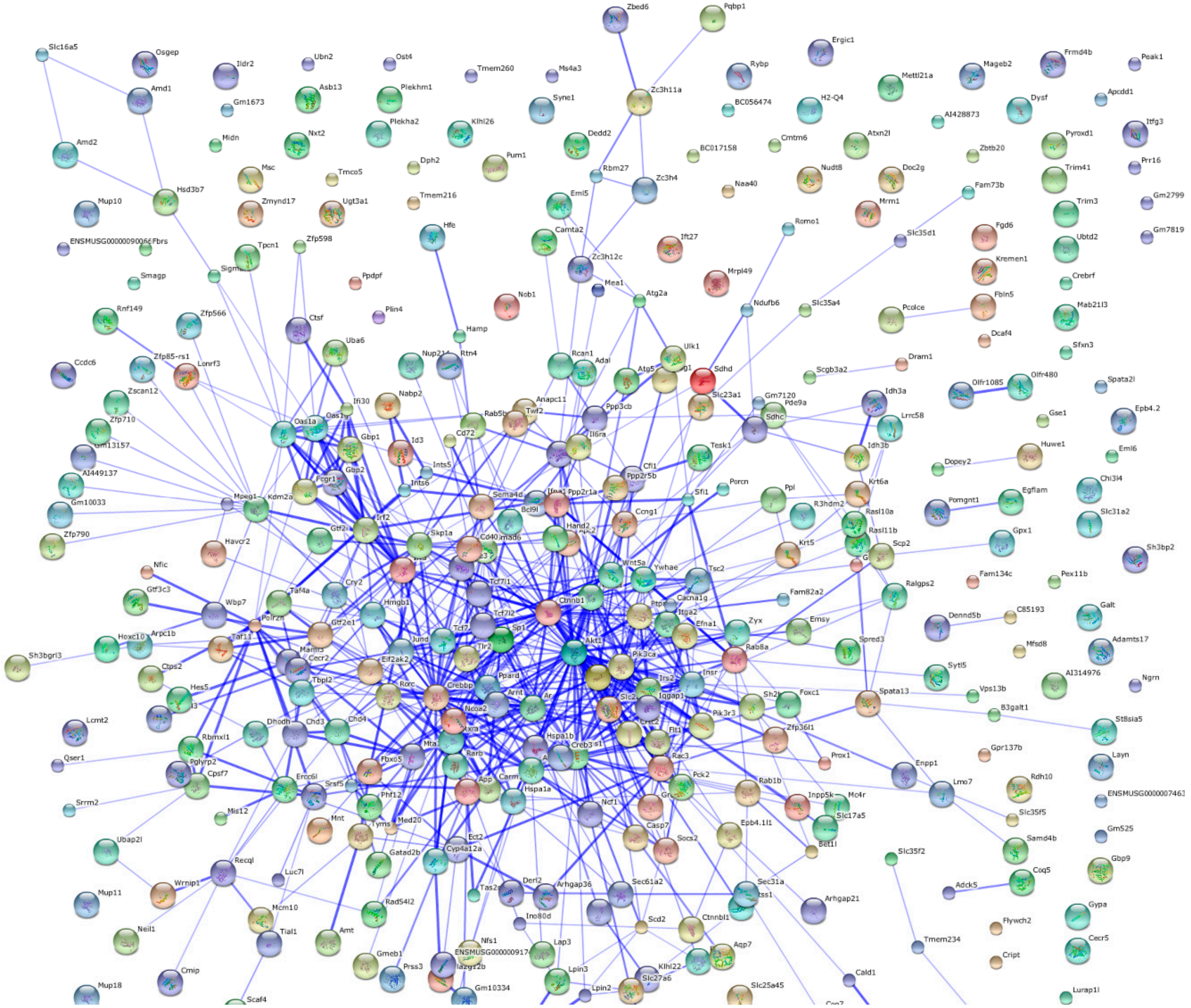

Figure S3. Interaction networks maps of differentially expressed genes. There were 13 nodes with ≥10 joint-edges in the map, including 190 joint-edges, which accounted for 88% of the total. Stronger associations are represented by thicker lines.

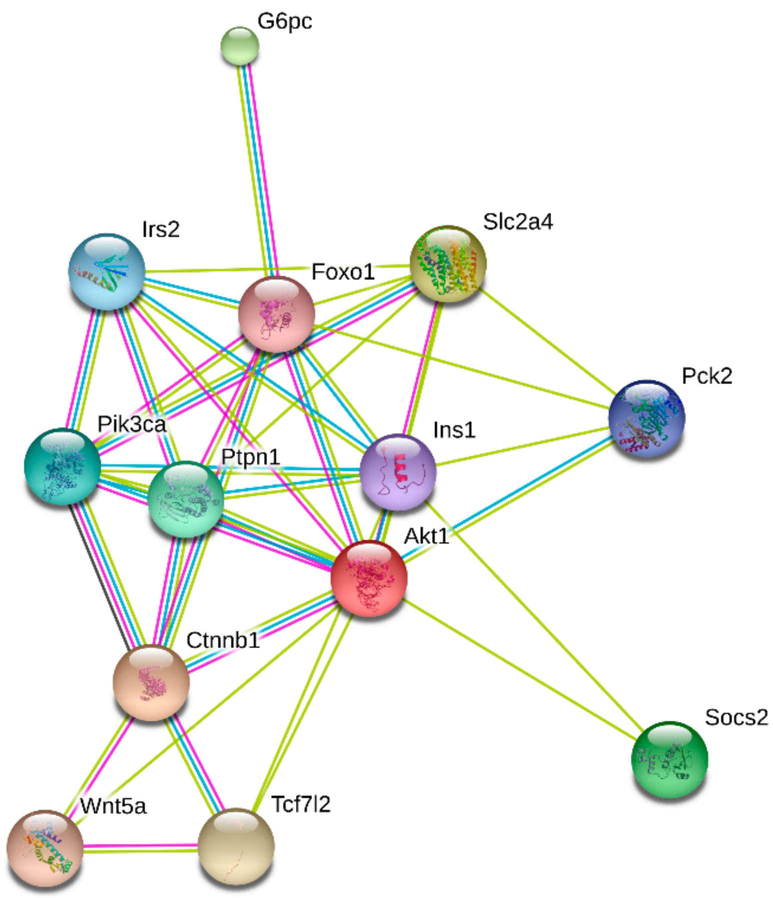

Figure S4. Interaction networks maps of differentially expressed genes in insulin signaling pathway and Wnt pathway. *Socs2*: suppressor of cytokine signaling 2; *Akt1*: Thymoma viral proto-oncogene 1; *Irs2*: insulin receptor substrate 2; *Pik3ca*: phosphatidylinositol 3-kinase, catalytic, alpha polypeptide; *Tcf7l2*: transcription factor 7 like 2; *Wnt5a*: wingless-type MMTV integration site family, member 5A; *Ctnnb1* (catenin, beta 1): catenin (cadherin associated protein), beta 1; *FoxO1*: forkhead box O1; *G6pc*: glucose-6-phosphatase, catalytic, *Ins1*: insulin 1; *Pck2* (*Pepck*): phosphoenolpyruvate carboxykinase 2; *Slc2a4* (*Glut4*): solute carrier family 2 member 4; *Ptpn1* (*Ptp1b*): protein tyrosine phosphatase, non-receptor type 1. Violet lines show experimentally determined interactions. Sky blue lines show known interactions from curated databases. Olive lines show interaction from text mining. Black lines show co-expression interaction.
